# Primary CD8^+^ T cells from elite suppressors effectively eliminate non-productively HIV-1 infected resting and activated CD4^+^ T cells

**DOI:** 10.1186/1742-4690-10-68

**Published:** 2013-07-01

**Authors:** Robert W Buckheit, Robert F Siliciano, Joel N Blankson

**Affiliations:** 1 Department of Medicine, Johns Hopkins University School of Medicine, 733 N. Broadway, BRB 880, Baltimore, MD 21205, USA; 2 Howard Hughes Medical Institute, Johns Hopkins University School of Medicine, 733 N. Broadway, Baltimore, MD 21205, USA

**Keywords:** HIV-1 Latency, Elite Suppression, CD8 Elimination, Non-productive Infection

## Abstract

**Background:**

Elite controllers or suppressors have the remarkable capacity to maintain HIV-1 plasma RNA levels below the limit of detection of clinical assays (<50 copies/mL) without therapy and have a lower frequency of latently infected cells compared to chronic progressors. While it is unclear how this reduced seeding of the reservoir is achieved, it is possible that effective CTL responses play an in important role in limiting the size of the latent reservoir.

**Results:**

Herein, we demonstrate that primary CD8^+^ T cells from HLA-B*57/5801 elite suppressors were able to efficiently eliminate resting and activated primary CD4^+^ T cells shortly after viral entry and prior to productive infection. CD8^+^ T cells from elite suppressors were significantly more effective at eliminating these cells than CD8^+^ T cells from chronic progressors.

**Conclusions:**

Nonproductively infected CD4^+^ T cells may represent a subpopulation of cells that are precursors to latently infected cells; therefore, the effective elimination of these cells may partially explain why elite suppressors have a much lower frequency of latently infected cells compared to chronic progressors. Thus, a vaccine strategy that elicits early and potent CD8^+^ T cell responses may have the capacity to limit the seeding of the latent reservoir in HIV-1 infection.

## Background

The development of a successful HIV-1 vaccine is paramount in combating the HIV-1 pandemic. In most patients, HIV-1 infection is characterized by high viral load and progressive CD4^+^ T cell decline. In these patients, known as chronic progressors (CP), AIDS develops in an average of ten years in the absence of antiretroviral therapy. Elite controllers or suppressors (ES) are remarkable HIV-1-infected individuals who restrict viral replication to below the limit of detection of standard clinical assays (<50 of HIV-1 RNA copies/mL of plasma) and represent less than one percent of the HIV-1 infected population [1,2]. The mechanism of this control is still unclear. A better understanding of the mechanisms involved in this control could be useful in defining the characteristics of an effective HIV-1 vaccine.

A qualitatively superior CD8^+^ T cell response has been most closely associated with control of viral replication. HLA-B*57 is over represented in ES cohorts [3-9] and was identified as a major determinant of control in multiple genome wide association studies [10-15]. Additionally, the maintenance of a polyfunctional HIV-1-specific CD8^+^ T cell response [16-18], as well as elevated proliferation and lytic granule loading have each been implicated in control of viral replication [3,19,20]. Unstimulated CD8^+^ T cell from ES have also been shown suppress viral replication more effectively compared to than CP CD8^+^ T cells [21,22]. Most convincingly, studies in the macaque model of elite suppression have shown that depletion of CD8^+^ T cells with monoclonal antibodies results in a loss of viral control [23,24].

While maintaining similar levels of circulating viremia as antiretroviral treated patients [3,25-27], ES have a reduced number of latently infected cells compared to CP [5,28-30]. A sensitive co-culture assay [31] was used to show that the frequency of latently infected cells in the blood of ES was 10 to 50 fold lower than the frequency observed for CPs who were on suppressive HAART regimens [29]. Additionally, ES have significantly lower levels of integrated proviral DNA compared to CP [28]. The lower levels of peak viremia during acute infection [32,33] that have been observed in some ES may partially explain the low frequency of latently infected cells in these patients. However, other mechanisms may contribute to this phenomenon. In a primary model system of latency, CD8^+^ T cells from ES were observed to be more effective at targeting reactivated latently infected cell than CD8^+^ T cells from individuals on suppressive HAART regimens [34]. Additionally, in a recent study in the SIV macaque model, a CMV based vaccine strategy was shown to provide impressive control of SIV replication in a subset of vaccinated animals. Interestingly, this control of viral replication was not abrogated when CD8^+^ T cell were depleted using antibodies, and very few SIV infected CD4^+^ T cells were seen at necropsy, suggesting that complete clearance of virus may have been achieved [35].

We hypothesized that ES CD8^+^ T cells might be capable of eliminating infected CD4^+^ T cells that are precursors of latently infected cells. Latency may be established when infected CD4^+^ T lymphoblasts revert back to a resting state that is non-permissive for viral gene expression [36] or through direct infection of resting CD4^+^ T cells [37]. In either case, the elimination of CD4^+^ T cells immediately after infection and before any viral genes are expressed should help prevent the establishment of latency. This targeting of non-productively infected cells early after viral infection by CD8^+^ T cell clones and cell lines has been documented in HIV and SIV infection models [38-40] however, it is unclear whether primary CD8^+^ T cells from ES can mediate this type of response.

In this study, we use a CD8^+^ T cell elimination assay with unstimulated, autologous CD4^+^ T cell targets to demonstrate that CD8^+^ T cells from HLA-B*57^+^ ES are able to eliminate non-productively infected resting and activated CD4^+^ T cells. This response was significantly superior to the response observed in both HLA-B*57/5801^+^ and HLA-B*57/5801^-^ CPs. Therefore, a potent CD8^+^ T cell response that targets non-productively infected cells in ES could contribute to the control of viral replication and may partially explain the reduced frequency of latently infected CD4^+^ T cells.

## Results and discussion

**Figure 1:**
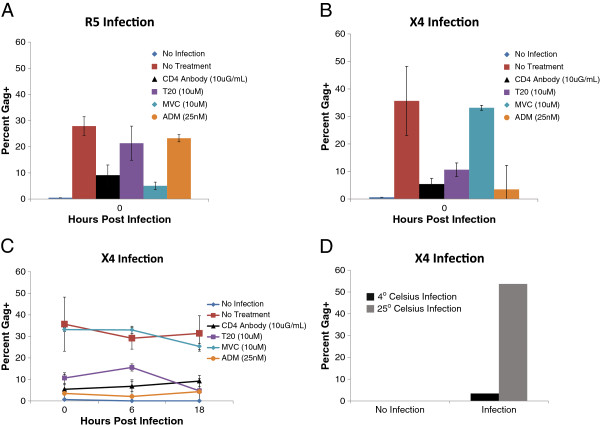
**Early Gag positivity in spinoculated cells is CD4 and co-receptor dependant.** CD4^+^ T cells were either untreated or treated with anti-CD4 antibody, MVC, ADM, or T20 for the duration of spinoculation. Percent Gag^+^ cells were analyzed immediately after spinoculation with R5 **(A)** or X4 **(B)** pseuotype virus. **(C)** X4 virus infection was analyzed at 0, 6 and 18 hours post spinoculation after treatment with anti-CD4 antibody, MVC, ADM, or T20 for the duration of the culture period. **(D)** Comparision of the percent infection 24 hours after spinoculation with and without T20 treatment (n=3). Standard error of the mean are indicated by black bars.

To measure infection immediately after spinoculation, we infected CD4^+^ T cells and stained with an antibody against the Gag p24 antigen. This allowed for detection of Gag stained cells shortly after infection, whereas GFP can only be reliably detected two to three days after infection. To observe the kinetics of infection, we analyzed infection in 3 healthy donors. We spinoculated primary CD4+ T cells with both an X4 and R5 pseudotyped virus, and treated the cells with an antibody specific for the CD4 receptor, 1.2 μM maraviroc (MVC), 25 nM ADM3100 (ADM) or 10 μM T20 during spinoculation. Gag staining was then performed immediately after spinoculation (hour 0). The CD4 antibody should block both X4 and R5 viruses from binding the primary receptor CD4, whereas MVC and ADC are specific antagonist of CCR5 and CXCR4 and should inhibit the infection of R5 and X4 viruses, respectively. Treatment with anti-CD4 antibody reduced infection at 0 hours post infection in for both R5 and X4 pseudotype viruses. MVC treatment specifically inhibited R5 virus infection (Figure 1A), whereas ADM specifically inhibited X4 virus infection (Figure 1B). Thus, the high level of Gag positivity seen immediately after spinoculation is mediated by virus binding that is CD4 and co-receptor specific. We also treated CD4^+^ T cells with T20 during spinoculation and observed a reduction in the level of Gag positivity suggesting that viral fusion and entry occurs during this process (Figure 1A, B). However, this was an incomplete block suggesting that some of the Gag positivity observed immediately after spinoculation could represent virus that has bound the cell surface, but has yet to fuse with and enter the cell.

To characterize the kinetics of infection for the X4 pseudotype virus, we observed infection at 0, 6, and 18 hours post spinoculation. Infection remained relatively constant from hour 0 to hour 18, and T20, CD4, and ADC treatment maintained a reduction in the level of infection (Figure 1C). Additionally, spinoculation with X4 virus was performed at 4° Celsius and 25° Celsius and little Gag positivity was observed by FACS analysis at the lower temperature (Figure 1D). Thus, spinoculation is resulting in a high level of interaction between virion and target cells that is CD4 and co-receptor specific, and largely requires fusion of the bound virion. To determine if the ES CD8^+^ T cell response to non-productively infected CD4^+^ T cells is superior to the response of CP CD8^+^ T cells, a variant of the CD8^+^ T cell suppression assay was used [21,22,41]. Freshly isolated, unstimulated CD4^+^ T cells were infected with 1000 ng of NL4-3 ΔEnv/GFP per 10^6^ cells with or without treatment with 10 μM EFV, and co-cultured with either Gag-stimulated or unstimulated CD8^+^ T cells at E:T ratios ranging from 1:1 to 1:8. CD8^+^ T cell effectors were added to the culture immediately after infection. The percent of cells with intracellular Gag was determined at 6, 18, and 72 hours after infection to measure the kinetics of the CD8^+^ T cell response.

The HLA-B*57 and HLA-B*5801 alleles are over-represented in ES cohorts cohorts [3-9], and HLA-B*57 is the major genetic determinant of control of HIV-1 replication as identified in multiple genome wide association studies [10-14]. Additionally, HLA-B*57 is thought to present peptides from structurally conserved regions of Gag, and mutations in these Gag epitopes have been associated with viral attenuation [42,43]. Therefore, we asked whether CD8^+^ T cells from HLA-B*57/5801^+^ ES were able to target proteins from the incoming virion, prior to the productive infection of target CD4^+^ T cells. Four experimental groups were analyzed: HLA-B*57^+^/ HLA-B*5801^+^ ES (n=10), HLA-B*57/5801^+^ CP (n=9), HLA-B*57/5801- CP (n=8) and healthy donors (HD) (n=6). Because the HLA-B*57/5801^+^ alleles are thought to present structurally conserved regions in Gag, we analyzed both HLA-B*57/5801^+^ CP and HLA-B*57/5801- CP to determine if there was a difference in the kinetics of the immune response if Gag was specifically targeted.

**Figure 2:**
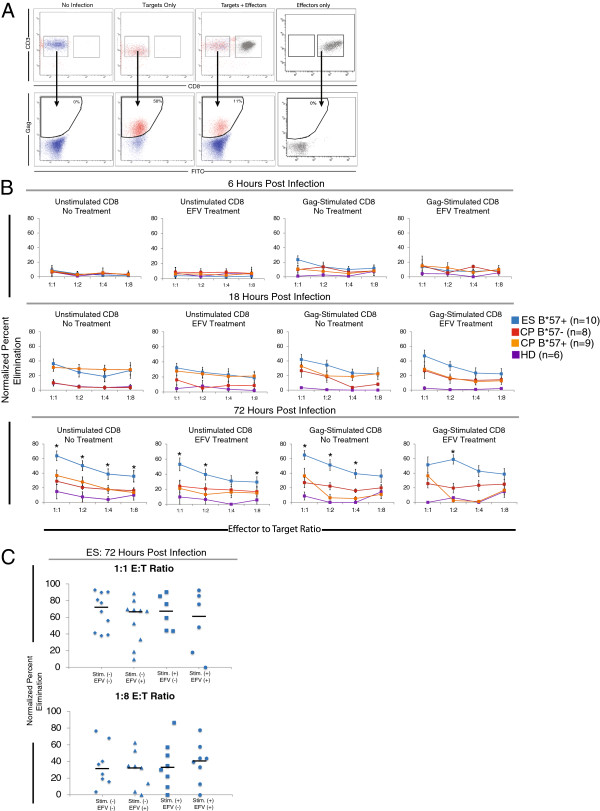
**Elimination of non-productively infected CD4**^**+ **^**cells. (A)** Representative FACS plots demonstrating the gating scheme employed for the calculation of normalized percent elimination. Cells in culture were stained with anti-CD3 and anti-CD8 antibodies to distinguish targets (CD3^+^/CD8-) and effector (CD3^+^/CD8^+^) populations. Target cells were then gated to determine the percent of gag positive cells, as determined by intracellular staining with an anti-Gag antibody. Uninfected target cells were used as a negative control. **(B)** An elimination assay was performed to determine the ability of CD8 T cells from B*57/5801^+^ ES (n=10; blue squares), B*57/5801^+^CP (n=9; orange squares), B*57/5801- CP (n=8; red squares) and healthy donors (HD, n=6; purple squares) to reduce the frequency of Gag positive target cells. Unstimulated CD8^+^ T cells or Gag Stimulated CD8^+^ T cells were co-cultured with untreated or EFV treated, autologous CD4^+^ T cell targets at various effector to target ratios. Elimination was analyzed after 6, 18 and 72 hours post infection. Data points where the level of elimination mediated by the ES CD8 T cells was significantly higher than all other experimental groups are indicated (black asterisks, p<.05). **(C)** The normalized percent elimination for ES Gag-stimulated and unstimulated effectors, for both untreated and 10 μM EFV treated were analyzed at 72 hours post infection for a 1:1 and 1:8 effector to target ratio. For all treatment groups, no statistical difference in the levels of elimination was observed. Median elimination levels are indicated.

Figure 2A shows representative FACS plots to illustrate the gating scheme. HIV-1 infection results in Nef-mediated CD4 downregulation [44]; therefore, target CD4^+^ T cells were designated as CD3^+^/CD8^-^ and then assayed for the expression of Gag (Figure 2A). The percent elimination was normalized based on the positive control values observed in absence of CD8^+^ effector cells. Gag staining can be reliably detected early after spinoculation, and prior to the detection of GFP expression, which requires *de novo* synthesis of proteins encoded on the viral genome. Similar levels of Gag positivity were detected between the ES (49.1% mean Gag positivity) and both CP groups (51.2% and 52.1 mean Gag positivity for B*57/5801^+^ CP and B*57/5801^-^ CP, respectively, data not shown). After 6 hours of co-culture, there were no significant differences between the experimental groups in the levels of elimination at any E:T ratio analyzed (Figure 2B). An increased level of elimination was observed for all experimental groups after 18 hours of infection. The levels of elimination mediated by Gag-peptide stimulated CD8^+^ T cells from ES was highest at a 1:1 E:T ratio for both EFV-treated and untreated cells, but did not reach statistical significance between these treatment groups. There was no difference in the level of elimination observed for untreated or EFV-treated CD4^+^ T cell targets. After 72 hours of infection, there was significantly more elimination by ES CD8^+^ T cells compared to CD8^+^ T cells from CP or HD. This increased elimination was observed for both unstimulated and Gag-stimulated CD8^+^ T cells and was similar when either EFV-treated or untreated CD4^+^ targets were used (Figure 2B). Gag peptide stimulation did not dramatically increase the elimination mediated by CD8^+^ T cells from either HLA-B*57/5801^+^ CP or HLA-B*57/5801- CP when compared to unstimulated CD8^+^ T cell effectors. This may be due to the fact that CD8^+^ T cells from CP undergo limited proliferation [19] and lytic granule loading [3] after stimulation with HIV peptides. No GFP expression was observed for any EFV-treated sample during the first 72 hours after infection (data not shown).

We next asked whether the levels of elimination of non-productively infected cells was less than the elimination of productively infected CD4^+^ T cells. Infection of CD4^+^ T cells in the presence of 10 μM EFV inhibits viral reverse transcription, and results in non-productively infected cells. For the ES group, the level of elimination of untreated or EFV-treated cells mediated by Gag-stimulated or unstimulated CD8^+^ T cells was analyzed 72 hours after infection at a 1:1 E:T ratio and a 1:8 E:T ratio (Figure 1C). No statistically significant difference between the levels of elimination of EFV-treated or untreated CD4^+^ T cell targets was observed for either Gag-stimulated or unstimulated CD8^+^ T cells.

**Figure 3:**
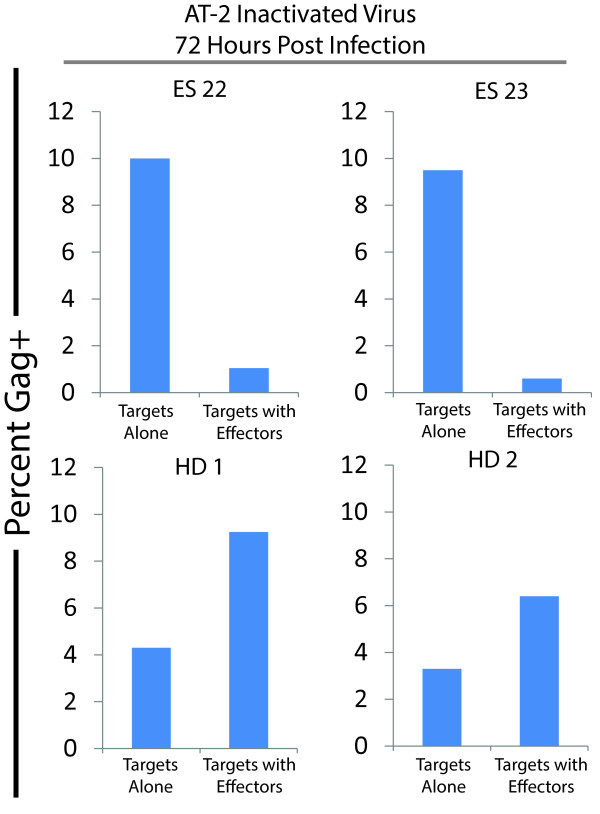
**Elimination of cells infected with inactivated virus.** CD4^+^ T cells from two ES (top panels) and two HD (bottom panels) were infected with AT-2 inactivated virus, and the level of Gag positivity was analyzed for target alone wells, and targets with effectors at a 1:1 effector to target ratio. No elimination was mediated by CD8 T cells from HD, in contrast to high level of elimination observed for the two ES assayed.

We confirmed this elimination of non-productively infected CD4^+^ T cells by infecting CD4^+^ T cells with virus inactivated by treatment with 300 μM aldirithiol-2 (AT-2). This agent has been shown to modify the essential zinc finger domains of HIV-1 nucleocapsid, thus, completely inactivate the virus while maintaining the structure of surface proteins. This allows the virus to enter target cells but does not allow the virus to replicate [45]. We confirmed this by demonstrating the presence of cell-associated Gag protein but not GFP expression after infection (data not shown). ES CD8^+^ T cells effectively eliminated CD4^+^ T cells infected with AT-2 treated virus, confirming the fact that these CTL can eliminated non-productively infected CD4^+^ T cells (Figure 3).

**Figure 4:**
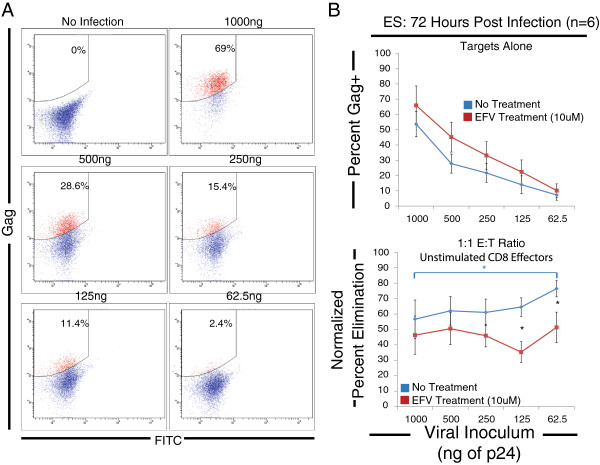
**Viral elimination is constant over a range of inoculum sizes. (A)** Representative FACS plots showing the titration of gag positivity from a viral inoculum size of 1000 ng to a viral inoculum size of 62.5 ng per one million cells. **(B)** The average percent infection as a result of viral titration was calculated over a range of viral inoculum sizes in a subset of our ES cohort (n=6; top panel). Unstimulated CD8^+^ T cells were added to either untreated (Blue squares) or 10 μM EFV treated ( Red squares) CD4^+^ T cells targets at a 1:1 effector to target ratio and the level of elimination was calculated 72 hours after infection (bottom panel). Statistically significant differences in elimination between the viral inoculum size for the untreated CD4^+^ T cell target group is indicated (blue bracket and asterisks). Differences in elimination between the EFV treated and untreated treatment groups are indicated (black asterisks).

To determine how the amount of input virus affects elimination, we varied the input virus from 1000 ng p24 to 62.5 ng p24 per 10^6^ CD4^+^ T cells by two fold dilutions. We assayed the normalized percent elimination by unstimulated CD8^+^ T cells from 6 ES at a 1:1 E:T ratio after 72 hours after infection. This assay was performed on EFV-treated and untreated CD4^+^ T cell targets. Representative FACS plots showing the gating strategy and the percentage of infected cells over the titration range are shown in Figure 4A. There was no statistical difference in the level of Gag present in CD4^+^ T cell targets with or without EFV treatment. These results confirm that at all the virus input levels tested, the observed Gag-staining does not depend on completion of reverse transcription and likely reflects cell-associated Gag proteins on incoming virus particles bound to or internalized by target cells. The average percent Gag positivity ranged from 65.8 percent to 7.2 percent (Figure 4B, top panel). The normalized percent elimination observed for untreated and EFV-treated CD4^+^ targets remained constant over a range of inoculum sizes (Figure 4B, bottom panel). For the untreated cells, the only significant difference in the level of elimination was between the 1000 ng p24 and the 62.5 ng p24 inoculum size (Figure 4B, blue asterisk). In this instance, significantly higher elimination was observed for the lower inoculum size. For EFV treated targets, there were no statistically significant differences in the level of elimination observed at any viral inoculum size. The level of elimination observed at the 1000, 500, and 250 ng p24 inoculum sizes was not statistically different between the untreated and EFV treated targets. However, there was a significant difference between the elimination observed for the two lowest doses of input virus between the treated and untreated CD4^+^ T cell targets (Figure 4B, black asterisks), indicating that recognition of viral infection at lower inoculum sizes might be more dependent on endogenously produced viral proteins. These data indicate that the level of elimination of infected cells is only weakly dependent on the viral inoculum size.

**Figure 5:**
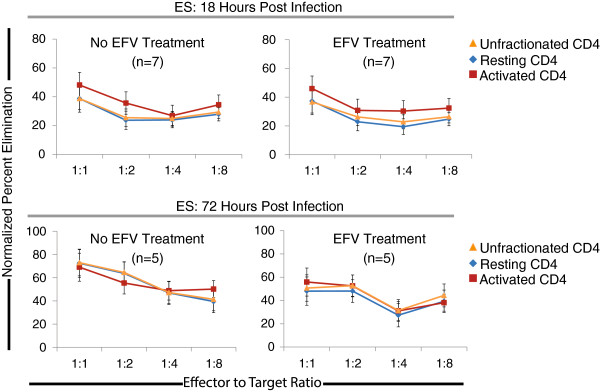
**Equal elimination of resting and activated CD4**^**+ **^**T cells.** In a subset of our ES cohort, target CD4^+^ T cells analyzed in the suppression assay were also stained with anti-HLA-DR, anti-CD25, and anti-CD69 antibodies and gated using the FACSDiva software to delineate resting and activated CD4^+^ cell populations. The level of elimination of untreated and EFV treated resting (blue diamonds), activated (red squares), and unfractionated (all CD3^+^/CD8- targets; orange circles) target cells was calculated at various effector to target ratios. This analysis was performed at 18 (top panels) and 72 hours (bottom panels) after infection. No statistically significant differences in the levels of elimination between each of the populations were observed.

We next asked whether CD8^+^ T cells from ES could eliminate both activated and resting target cells. Therefore, 72 hours after infection during analysis, we stained CD4^+^ target cells from ES with a cocktail of anti-HLA-DR, anti-CD25, and anti-CD69 antibodies in addition to anti-CD3 and anti-CD8 antibodies used to distinguish targets and effectors. Cells that were positive for one or more of these activation markers were considered to be activated CD4^+^ T cells, and those that were negative for all three of these markers were considered to be resting CD4^+^ T cells. There were no significant differences in the levels of resting and activated CD4^+^ T cells that were productively (GFP^+^) and non-productively (Gag^+^ GFP^-^) infected (Additional file 1). The non-productive infection of resting CD4+ T cells is consistent with a prior study that showed fusion of X4 pseudotyped virus to unstimulated CD4+ T cells [46]. The GFP expressing resting CD4^+^ T cells may represent cells that were recently activated but no longer express classical activation markers. This was not seen in a prior study when resting CD4^+^ T cells were purified prior to infection [47], thus it is possible that cytokines secreted by activated CD4^+^ T cells in the unfractionated collection of cells may have facilitated the productive infection of resting CD4^+^ T cells. We asked whether CD8^+^ T cells from ES could eliminate both of these cells types after 18 and 72 hours after infection, both with and without treatment with EFV. We also calculated the percent elimination of infected cells for unfractionated CD4^+^ targets (defined as all CD3^+^/CD8^-^ cells within the experimental well). Similar levels of elimination of both resting and activated infected CD4^+^ T cells were observed over a range of E:T ratios (Figure 5B). At both 18 and 72 hours after infection, there was no statistical difference between the levels of elimination of activated and resting infected CD4^+^ T cells, and no difference when compared to unfractionated CD4^+^ T cell targets. Additionally, there was no difference in the level of elimination of infected cells observed when cells were treated or not treated with 10 μM EFV.

Studies have shown that ES CD4^+^ T cells have lower levels of total [5,48] and integrated [28] HIV DNA, and replication-competent HIV-1 [29] compared to patients on suppressive HAART regimens. It is possible that precursors of latently infected cells are cells that were infected as they were transitioning from activated to a resting state, resulting in a non-productively infected cell. Therefore, if ES CD8^+^ T were able to effectively target non-productively infected cells through proteins from incoming virions, they would be able to reduce the seeding of the latent reservoir. By treating infected CD4^+^ T cells with EFV, and by infecting cells with AT-2 inactivated virus, we were able to produce non-productively infected cells. We were able to demonstrate equal elimination of infected cells between EFV treated and untreated cells and effective elimination of CD4^+^ T cells infected with AT-2 inactivated virus. This elimination of infected cells was relatively constant over a wide range of viral inoculum sizes, implying that in some instances, proteins from the infecting virion are sufficient to induce a CD8^+^ T cells response without additional viral replication. While levels of virus used in this study may be in excess of levels typically observed in peripheral blood, local concentrations of virus in the tissue and lymph nodes could be similar depending on the HIV-1 burst size as previously hypothesized [38].

While a recent study suggested that HIV-1-specific CD8^+^ T cell lines that recognized HLA-B*57 restricted epitopes were capable of eliminating non-productively CD4^+^ T cells [39], we show here that non-stimulated primary CD8^+^ T cells from HLA-B*57/5801 ES were significantly more effective at the elimination of infected CD4^+^ T cells than CD8^+^ T cells from HLA-B*57/5801 CP at 72 hours post infection. The reverse transcription process in resting CD4^+^ T cells has been shown to take 2–3 days [47], therefore, an increase in the elimination potential over this time frame could limit the seeding of the latent reservoir. Furthermore, non-productively infected CD4^+^ T cells would not be expected to die from the cytopathic effect of the virus, so even CTL responses that took 72 hours to mature would be effective at eliminating these cells.

It should be noted that ES are a heterogeneous population, and that not all ES possess previously reported, protective HLA alleles and, in some cases, have weak or absent immune responses [6,7,49,50]. Thus, it would be interesting to determine if ES without protective HLA types are also able to target non-productively infected cells.

While only HLA-B*57/5801^+^ ES were analyzed in this study, these data have implications for the design of an effective HIV-1 vaccine. CD8^+^ T cell vaccines that are able to elicit a potent response early in infection may have the potential to reduce the seeding of the latent reservoir. The CD8^+^ T cell responses described may partially explain the low frequency of latently infected cells that is observed in ES. The small reservoir size may be due to both the effective elimination of productively infected CD4^+^ T cells [3,16,18-22], which would limit the level of viremia and the subsequent seeding of the reservoir, and by the elimination of non-productively infected CD4^+^ T cells that may be the precursors of the latent reservoir. These data could also explain why ES maintain low frequencies of latently infected cells in chronic infection in spite of ongoing HIV-1 replication [51-55]; recently infected resting and activated CD4^+^ T cells will be eliminated regardless of whether productive or non-productive infection occurs.

## Conclusions

This is the first study to show effective elimination of non-productively infected CD4^+^ T cells by unstimulated primary HIV-1-specific CD8^+^ T cells from ES. The results suggest that an early and potent CD8^+^ T cell immune response could result in a lower baseline latent reservoir size as has been seen with a potent CD8^+^ T cell vaccine in the SIV model [35]. Latency remains the largest barrier to the eradication of HIV-1 infection, and understanding the mechanisms of an optimal CD8^+^ T cell response can allow for improved immune based eradication strategies.

## Methods

### Patients

All individuals provided written informed consent prior to participating in this study, and all studies were approved by the Johns Hopkins Institutional Review Board. All ES maintained undetectable plasma HIV-1 RNA levels for the duration of study (<50 copies/mL) and are positive for the HLA-B*57/5801 allele. The median CD4^+^ T cell count for the ES used in this study was 857 cells/ul (range 453–1638 cells/ul), and the median duration of infection was 13 years (range 4–28 years). All CP were on suppressive antiretroviral therapy for greater than a year at the time of study. Of the CP used for the study, 9 were positive for the HLA-B*57/5801 allele and 8 were negative for the HLA-B*57/5801 allele. Seronegative controls were 6 healthy laboratory workers (HD, healthy donors).

### Isolation of CD4^+^ and CD8^+^ T cells

PBMCs were isolated from whole blood by ficoll gradient centrifugation. CD8^+^ T cells were isolated by positive selection using Human CD8 Microbeads following the manufacturer’s guidelines (Miltenyi Biotec). CD4^+^ T cell were subsequently isolated by negative selection using the Human CD4 T cell isolation kit II following the manufacture’s guidelines (Miltenyi Biotech). All cells were maintained in non-stimulating media (RPMI ^+^ 10% FBS without exogenous cytokines) for the duration of experimentation unless otherwise noted.

### Infection

An NL4-3 pseudovirus with GFP in the place of Env (NL4-3 ΔEnv/GFP) was used for all infections, as previously described [56]. The virus was produced by cotransfection of 239 T cells with the proviral construct pNL4-3 ΔEnv/GFP and either an R5 or X4 Env expression vector to provide the Env in trans. The *env* expression vectors have been routinely used by our lab group [57]. CD4^+^ T cells were infected by spinoculation as previously described [58]. Briefly, CD4^+^ T cells were isolated and collected in 50 mL conical tubes. The cells were pelleted by centrifugation at 1200 RPM for 10 minutes, and all supernatant was removed. The cells were then resuspended in the viral inoculum. Spinoculation was performed by spinning at 1200 × g for 2 hours at room temperature. Cells were then resuspended at a concentration of 1 × 10^6^ cells/mL and plated at 1 × 10^5^ cells/well in a 96 well, round bottom plate. CD4^+^ T cells were not activated prior to spinoculation. Rather they were isolated directly from donors and maintained in non-stimulating media for the duration of the experiment as previously described [57,59]. CD4^+^ T cells were infected with a viral inoculum that routinely resulted in approximately 15-20% infection after 72 hours post infection, as measured by GFP expression (1000 ng p24/1e6 cells). For titration experiments, the level of virus was titrated by two fold dilutions from 1000 ng p24/10^6^ cells to 62.5 ng p24/10^6^ cells.

For experiments involving efavirenz (EFV) treatment, EFV was added to CD4^+^ T cells at the time of spinoculation and maintained in the culture for the duration of the experiment at 10 μM which is greater than the IC_99_. Treatment of CD4^+^ T cells with EFV prevented any expression of GFP after 72 hours of infection in any of the treated wells (data not shown). An aliquot of uninfected CD4^+^ T cells was kept as a negative control.

For experiments involving maraviroc (MVC), ADM3100 (ADM) and T20, drug was added to CD4^+^ T cells at the time of spinoculation and maintained in the culture for the duration of the experiment at 10 μM, 10 μM and 25 nM, respectively, which is greater than the IC_99_.

AT-2 inactivated virus was produced as previously described [45]. Briefly, NL4-3 ΔEnv/GFP virus was treated with 300 μM AT-2 (Sigma) for 1 hour at 37°C. After incubation, virus was filtered using Amicon Ultra 0.5 mL filtration tubes (Millipore). Virus was then kept on ice until use in the CD8^+^ suppression assay.

### CD8^+^ T Cell elimination of infected cells assay

The CD8^+^ T cell elimination assay was modified from a previously reported suppression assay [21,22]. We measured the reduction in the number of cells expressing Gag in the presence of CD8+ T cells. Because we used a single cycle virus, this reduction was most likely due to killing of infected cells although we do not directly prove this in this study. To prepare Gag-stimulated CD8^+^ T cells, PBMCs from patients or HD were isolated one week prior to the execution of the suppression assay (day −7). PBMCs were stimulated with a mixture of overlapping Gag peptides that spanned the length of Gag at a total concentration of 5 μg/mL and IL-2 (2 units/mL). After one week, Gag-stimulated, CD8^+^ effectors were isolated by positive selection using CD8 microbeads (Miltenyi Biotech). A second blood draw was obtained from each patient or HD on the same day, and unstimulated CD8^+^ T cell effectors and autologous CD4^+^ T cell targets were isolated directed *ex vivo*, by positive and negative selection, respectively. CD8^+^ T cells were added to infected CD4^+^ T cell targets immediately after spinoculation. The infected CD4^+^ targets, with or without EFV treatment, and either Gag-stimulated or unstimulated CD8 effectors were co-cultured in a 96 well plate at varying effector to target (E:T) ratios. The number of CD4^+^ T cells per well remained constant (100,000 cells per well), and the number of CD8^+^ T cells was varied. The cells were cultured in a final volume of 200 μL of non-stimulating media. CD8^+^ T cells were serially diluted from a 1:1 effector to target (E:T) ratio to a 1:8 E:T ratio by two-fold dilutions. Wells with only CD4^+^ T cells (targets alone) were used as positive controls for normalization.

Elimination of infected cells was measured at 6, 18 and 72 hours after infection to determine the kinetics of the response. To measure infection, cells were stained with anti-CD3 Pac Blue (Becton Dickinson), anti-CD8 APC-H7 (Becton Dickinson), and anti-Gag PE (Beckmen Colter, coulter clone kc57). For intracellular staining, the Cytofix/Cytoperm was solution was used following the manufacturers guidelines (Becton Dickinson). The normalized percent elimination of infected cells was calculated as follows: (Percent Gag^+^ cells in wells with Targets alone – Percent of CD4^+^ T cells that are Gag^+^ in wells with Targets and Effectors) / (Percent Gag^+^ cells in wells with Targets alone) × 100, as described previously [41].

For the titration experiments, CD4^+^ T cells were infected with various viral inoculums, and cultured with our without unstimulated CD8^+^ T cells at a 1:1 E:T ratios. Cultures without CD8^+^ effectors served as the positive control for normalization.

For a subset of ES, cells were also stained with a cocktail of anti-HLA-DR APC, anti-CD25 APC, and anti-CD69 APC (Becton Dickinson). CD4^+^ T cell targets that expressed any one of these markers were designated activated CD4^+^ T cells, those that were negative for all three markers were designated as resting CD4^+^ T cells, as gated using the FACS Diva software. The percent infection of unfractionated CD4^+^ T cells (all CD3^+^/CD8^-^ cells), resting CD4^+^ T cells and activated CD4^+^ T cells w calculated, and the normalized percent elimination of infected cells of each subset was calculated.

All cytometric analyses were performed using a FACS Canto II (Becton Dickinson) and analyzed using the FACS Diva software.

### Statistical analysis

For the analysis of the significance of the difference between populations, the Mann–Whitney nonparametric T test was used. For pair-wise comparison of untreated vs. EFV-treated cells, a paired Student’s T test was used. P values were calculated, and a P value of less than 0.05 was considered significant.

## Competing interests

The authors declare no competing financial conflicts of interest.

## Authors’ contributions

RWB carried out all experiments, and drafted the manuscript. RFS participated in the design and planning of experiments and helped to edit the manuscript. JNB conceived of the study, and participated in its design and coordination and helped to draft the manuscript. All authors read and approved the final manuscript.

## Supplementary Material

Additional file 1 Percent of Gag and GFP positive resting and activated cells present at 18 and 72 hours post infection in the presence or absence of EFV. Click here for file

## References

[B1] Blankson JN The study of elite controllers: a pure academic exercise or a potential pathway to an HIV-1 vaccine? Curr Opin HIV AIDS 2011 6 3 147 150 10.1097/COH.0b013e3283457868 21399493

[B2] Ockulicz J Lambotte O 1. Epidemiology and clinical characteristics of elite controllers Current Opinions on HIV AIDS 2011 6 3 163 168 10.1097/COH.0b013e328344f35e 21502920

[B3] Migueles SA Osborne CM Royce C Compton AA Joshi RP Weeks KA Rood JE Berkley AM Sacha JB Cogliano-Shutta NA Lloyd M Roby G Kwan R McLaughlin M Stallings S Rehm C O'Shea MA Mican J Packard BZ Komoriya A Palmer S Wiegand AP Maldarelli F Coffin JM Mellors JW Hallahan CW Follman DA Connors M Lytic granule loading of CD8+ T cells is required for HIV-infected cell elimination associated with immune control Immunity 2008 29 6 1009 1021 10.1016/j.immuni.2008.10.010 19062316 PMC2622434

[B4] Migueles SA Sabbaghian MS Shupert WL Bettinotti MP Marincola FM Martino L Hallahan CW Selig SM Schwartz D Sullivan J Connors M HLA B*5701 is highly associated with restriction of virus replication in a subgroup of HIV-infected long term nonprogressors Proc Natl Acad Sci USA 2000 97 6 2709 2714 10.1073/pnas.050567397 10694578 PMC15994

[B5] Lambotte O Boufassa F Madec Y Nguyen A Goujard C Meyer L Rouzioux C Venet A Delfraissy JF SEROCO-HEMOCO Study Group HIV controllers: a homogeneous group of HIV-1-infected patients with spontaneous control of viral replication Clin Infect Dis 2005 41 7 1053 1056 10.1086/433188 16142675

[B6] Emu B Sinclair E Hatano H Ferre A Shacklett B Martin JN McCune JM Deeks SG HLA class I-restricted T-cell responses may contribute to the control of human immunodeficiency virus infection, but such responses are not always necessary for long-term virus control J Virol 2008 82 11 5398 5407 10.1128/JVI.02176-07 18353945 PMC2395228

[B7] Pereyra F Addo MM Kaufmann DE Liu Y Miura T Rathod A Baker B Trocha A Rosenberg R Mackey E Ueda P Lu Z Cohen D Wrin T Petropoulos CJ Rosenberg ES Walker BD Genetic and immunologic heterogeneity among persons who control HIV infection in the absence of therapy J Infect Dis 2008 197 4 563 571 10.1086/526786 18275276

[B8] Han Y Lai J Barditch-Crovo P Gallant JE Williams TM Siliciano RF Blankson JN The role of protective HCP5 and HLA-C associated polymorphisms in the control of HIV-1 replication in a subset of elite suppressors AIDS 2008 22 4 541 544 10.1097/QAD.0b013e3282f470e4 18301071

[B9] Sajadi MM Constantine NT Mann DL Charurat M Dadzan E Kadlecik P Redfield RR Epidemiologic characteristics and natural history of HIV-1 natural viral suppressors J Acquir Immune Defic Syndr 2009 50 4 403 408 10.1097/QAI.0b013e3181945f1e 19214118 PMC2697612

[B10] Fellay J Shianna KV Ge D Colombo S Ledergerber B Weale M Zhang K Gumbs C Castagna A Cossarizza A Cozzi-Lepri A De Luca A Easterbrook P Francioli P Mallal S Martinez-Picado J Miro JM Obel N Smith JP Wyniger J Descombes P Antonarakis SE Letvin NL McMichael AJ Haynes BF Telenti A Goldstein DB A whole-genome association study of major determinants for host control of HIV-1 Science 2007 317 5840 944 947 10.1126/science.1143767 17641165 PMC1991296

[B11] Pereyra F Jia X McLaren PJ Telenti A de Bakker PI Walker BD Ripke S Brumme CJ Pulit SL Carrington M Kadie CM Carlson JM Heckerman D Graham RR Plenge RM Deeks SG Gianniny L Crawford G Sullivan J Gonzalez E Davies L Camargo A Moore JM Beattie N Gupta S Crenshaw A Burtt NP Guiducci C Gupta N Gao X International HIV Controllers Study The major genetic determinants of HIV-1 control affect HLA class I peptide presentation Science 2010 330 6010 1551 1557 21051598 10.1126/science.1195271 PMC3235490

[B12] Catano G Kulkarni H He W Marconi VC Agan BK Landrum M Anderson S Delmar J Telles V Song L Castiblanco J Clark RA Dolan MJ Ahuja SK HIV-1 disease-influencing effects associated with ZNRD1, HCP5 and HLA-C alleles are attributable mainly to either HLA-A10 or HLA-B*57 alleles PLoS One 2008 3 11 e3636 10.1371/journal.pone.0003636 18982067 PMC2574440

[B13] Dalmasso C Carpentier W Meyer L Rouzioux C Goujard C Chaix ML Lambotte O Avettand-Fenoel V Le Clerc S de Senneville LD Deveau C Boufassa F Debre P Delfraissy JF Broet P Theodorou I ANRS Genome Wide Association 01 Distinct genetic loci control plasma HIV-RNA and cellular HIV-DNA levels in HIV-1 infection: the ANRS Genome Wide Association 01 study PLoS One 2008 3 12 e3907 10.1371/journal.pone.0003907 19107206 PMC2603319

[B14] Limou S Le Clerc S Coulonges C Carpentier W Dina C Delaneau O Labib T Taing L Sladek R Deveau C Ratsimandresy R Montes M Spadoni JL Lelievre JD Levy Y Therwath A Schachter F Matsuda F Gut I Froguel P Delfraissy JF Hercberg S Zagury JF ANRS Genomic Group Genomewide association study of an AIDS-nonprogression cohort emphasizes the role played by HLA genes (ANRS Genomewide Association Study 02) J Infect Dis 2009 199 3 419 426 10.1086/596067 19115949

[B15] van Manen D Kootstra NA Boeser-Nunnink B Handulle MA van't Wout AB Schuitemaker H Association of HLA-C and HCP5 gene regions with the clinical course of HIV-1 infection AIDS 2009 23 1 19 28 10.1097/QAD.0b013e32831db247 19050382

[B16] Betts MR Nason MC West SM De Rosa SC Migueles SA Abraham J Lederman MM Benito JM Goepfert PA Connors M Roederer M Koup RA HIV nonprogressors preferentially maintain highly functional HIV-specific CD8+ T cells Blood 2006 107 12 4781 4789 10.1182/blood-2005-12-4818 16467198 PMC1895811

[B17] Almeida JR Price DA Papagno L Arkoub ZA Sauce D Bornstein E Asher TE Samri A Schnuriger A Theodorou I Costagliola D Rouzioux C Agut H Marcelin AG Douek D Autran B Appay V Superior control of HIV-1 replication by CD8+ T cells is reflected by their avidity, polyfunctionality, and clonal turnover J Exp Med 2007 204 10 2473 2485 10.1084/jem.20070784 17893201 PMC2118466

[B18] Ferre AL Hunt PW Critchfield JW Young DH Morris MM Garcia JC Pollard RB Yee HF Jr Martin JN Deeks SG Shacklett BL Mucosal immune responses to HIV-1 in elite controllers: a potential correlate of immune control Blood 2009 113 17 3978 3989 10.1182/blood-2008-10-182709 19109229 PMC2673124

[B19] Migueles SA Laborico AC Shupert WL Sabbaghian MS Rabin R Hallahan CW Van Baarle D Kostense S Miedema F McLaughlin M Ehler L Metcalf J Liu S Connors M HIV-specific CD8+ T cell proliferation is coupled to perforin expression and is maintained in nonprogressors Nat Immunol 2002 3 11 1061 1068 10.1038/ni845 12368910

[B20] Hersperger AR Pereyra F Nason M Demers K Sheth P Shin LY Kovacs CM Rodriguez B Sieg SF Teixeira-Johnson L Gudonis D Goepfert PA Lederman MM Frank I Makedonas G Kaul R Walker BD Betts MR Perforin expression directly ex vivo by HIV-specific CD8 T-cells is a correlate of HIV elite control PLoS Pathog 2010 6 5 e1000917 10.1371/journal.ppat.1000917 20523897 PMC2877741

[B21] Saez-Cirion A Sinet M Shin SY Urrutia A Versmisse P Lacabaratz C Boufassa F Avettand-Fenoel V Rouzioux C Delfraissy JF Barre-Sinoussi F Lambotte O Venet A Pancino G ANRS EP36 HIV Controllers Study Group Heterogeneity in HIV suppression by CD8 T cells from HIV controllers: association with Gag-specific CD8 T cell responses J Immunol 2009 182 12 7828 7837 10.4049/jimmunol.0803928 19494307

[B22] Saez-Cirion A Lacabaratz C Lambotte O Versmisse P Urrutia A Boufassa F Barre-Sinoussi F Delfraissy JF Sinet M Pancino G Venet A Agence Nationale de Recherches sur le Sida EP36 HIV Controllers Study Group HIV controllers exhibit potent CD8 T cell capacity to suppress HIV infection ex vivo and peculiar cytotoxic T lymphocyte activation phenotype Proc Natl Acad Sci USA 2007 104 16 6776 6781 10.1073/pnas.0611244104 17428922 PMC1851664

[B23] Friedrich TC Valentine LE Yant LJ Rakasz EG Piaskowski SM Furlott JR Weisgrau KL Burwitz B May GE Leon EJ Soma T Napoe G Capuano SV 3rd Wilson NA Watkins DI Subdominant CD8+ T-cell responses are involved in durable control of AIDS virus replication J Virol 2007 81 7 3465 3476 10.1128/JVI.02392-06 17251286 PMC1866056

[B24] Pandrea I Gaufin T Gautam R Kristoff J Mandell D Montefiori D Keele BF Ribeiro RM Veazey RS Apetrei C Functional cure of SIVagm infection in rhesus macaques results in complete recovery of CD4+ T cells and is reverted by CD8+ cell depletion PLoS Pathog 2011 7 8 e1002170 10.1371/journal.ppat.1002170 21829366 PMC3150280

[B25] Pereyra F Palmer S Miura T Block BL Wiegand A Rothchild AC Baker B Rosenberg R Cutrell E Seaman MS Coffin JM Walker BD Persistent low-level viremia in HIV-1 elite controllers and relationship to immunologic parameters J Infect Dis 2009 200 6 984 990 10.1086/605446 19656066 PMC3725728

[B26] Dinoso JB Kim SY Siliciano RF Blankson JN A comparison of viral loads between HIV-1-infected elite suppressors and individuals who receive suppressive highly active antiretroviral therapy Clin Infect Dis 2008 47 1 102 104 10.1086/588791 18494606 PMC2564994

[B27] Hatano H Delwart EL Norris PJ Lee TH Dunn-Williams J Hunt PW Hoh R Stramer SL Linnen JM McCune JM Martin JN Busch MP Deeks SG Evidence for persistent low-level viremia in individuals who control human immunodeficiency virus in the absence of antiretroviral therapy J Virol 2009 83 1 329 335 10.1128/JVI.01763-08 18945778 PMC2612329

[B28] Graf EH Mexas AM Yu JJ Shaheen F Liszewski MK Di Mascio M Migueles SA Connors M O'Doherty U Elite suppressors harbor low levels of integrated HIV DNA and high levels of 2-LTR circular HIV DNA compared to HIV+ patients on and off HAART PLoS Pathog 2011 7 2 e1001300 10.1371/journal.ppat.1001300 21383972 PMC3044690

[B29] Blankson JN Bailey JR Thayil S Yang HC Lassen K Lai J Gandhi SK Siliciano JD Williams TM Siliciano RF Isolation and characterization of replication-competent human immunodeficiency virus type 1 from a subset of elite suppressors J Virol 2007 81 5 2508 2518 10.1128/JVI.02165-06 17151109 PMC1865922

[B30] Julg B Pereyra F Buzon MJ Piechocka-Trocha A Clark MJ Baker BM Lian J Miura T Martinez-Picado J Addo MM Walker BD Infrequent recovery of HIV from but robust exogenous infection of activated CD4(+) T cells in HIV elite controllers Clin Infect Dis 2010 51 2 233 238 10.1086/653677 20550452 PMC3749734

[B31] Siliciano JD Siliciano RF Enhanced culture assay for detection and quantitation of latently infected, resting CD4+ T-cells carrying replication-competent virus in HIV-1-infected individuals Methods Mol Biol 2005 304 3 15 16061962 10.1385/1-59259-907-9:003

[B32] Altfeld M Addo MM Rosenberg ES Hecht FM Lee PK Vogel M Yu XG Draenert R Johnston MN Strick D Allen TM Feeney ME Kahn JO Sekaly RP Levy JA Rockstroh JK Goulder PJ Walker BD Influence of HLA-B57 on clinical presentation and viral control during acute HIV-1 infection AIDS 2003 17 18 2581 2591 10.1097/00002030-200312050-00005 14685052

[B33] Goujard C Chaix ML Lambotte O Deveau C Sinet M Guergnon J Courgnaud V Rouzioux C Delfraissy JF Venet A Meyer L Agence Nationale de Recherche sur le Sida PRIMO Study Group Spontaneous control of viral replication during primary HIV infection: when is "HIV controller" status established? Clin Infect Dis 2009 49 6 982 986 10.1086/605504 19681706

[B34] Shan L Deng K Shroff NS Durand CM Rabi SA Yang HC Zhang H Margolick JB Blankson JN Siliciano RF Stimulation of HIV-1-Specific Cytolytic T Lymphocytes Facilitates Elimination of Latent Viral Reservoir after Virus Reactivation Immunity 2012 36 3 491 501 10.1016/j.immuni.2012.01.014 22406268 PMC3501645

[B35] Hansen SG Ford JC Lewis MS Ventura AB Hughes CM Coyne-Johnson L Whizin N Oswald K Shoemaker R Swanson T Legasse AW Chiuchiolo MJ Parks CL Axthelm MK Nelson JA Jarvis MA Piatak M Jr Lifson JD Picker LJ Profound early control of highly pathogenic SIV by an effector memory T-cell vaccine Nature 2011 473 7348 523 527 10.1038/nature10003 21562493 PMC3102768

[B36] Yang HC Xing S Shan L O'Connell K Dinoso J Shen A Zhou Y Shrum CK Han Y Liu JO Zhang H Margolick JB Siliciano RF Small-molecule screening using a human primary cell model of HIV latency identifies compounds that reverse latency without cellular activation J Clin Invest 2009 119 11 3473 3486 19805909 10.1172/JCI39199 PMC2769176

[B37] Pace MJ Graf EH Agosto LM Mexas AM Male F Brady T Bushman FD O'Doherty U Directly infected resting CD4+T cells can produce HIV Gag without spreading infection in a model of HIV latency PLoS Pathog 2012 8 7 e1002818 10.1371/journal.ppat.1002818 22911005 PMC3406090

[B38] Sacha JB Chung C Rakasz EG Spencer SP Jonas AK Bean AT Lee W Burwitz BJ Stephany JJ Loffredo JT Allison DB Adnan S Hoji A Wilson NA Friedrich TC Lifson JD Yang OO Watkins DI Gag-specific CD8+ T lymphocytes recognize infected cells before AIDS-virus integration and viral protein expression J Immunol 2007 178 5 2746 2754 17312117 10.4049/jimmunol.178.5.2746 PMC4520734

[B39] Kloverpris HN Payne RP Sacha JB Rasaiyaah JT Chen F Takiguchi M Yang OO Towers GJ Goulder P Prado JG Early Antigen Presentation of Protective HIV-1 KF11Gag and KK10Gag Epitopes from Incoming Viral Particles Facilitates Rapid Recognition of Infected Cells by Specific CD8+ T Cells J Virol 2013 87 5 2628 2638 10.1128/JVI.02131-12 23255798 PMC3571362

[B40] Buseyne F Le Gall S Boccaccio C Abastado JP Lifson JD Arthur LO Riviere Y Heard JM Schwartz O MHC-I-restricted presentation of HIV-1 virion antigens without viral replication Nat Med 2001 7 3 344 349 10.1038/85493 11231634

[B41] Buckheit RW 3rd Salgado M Silciano RF Blankson JN Inhibitory potential of subpopulations of CD8+ T cells in HIV-1-infected elite suppressors J Virol 2012 86 24 13679 13688 10.1128/JVI.02439-12 23055552 PMC3503034

[B42] Martinez-Picado J Prado JG Fry EE Pfafferott K Leslie A Chetty S Thobakgale C Honeyborne I Crawford H Matthews P Pillay T Rousseau C Mullins JI Brander C Walker BD Stuart DI Kiepiela P Goulder P Fitness cost of escape mutations in p24 Gag in association with control of human immunodeficiency virus type 1 J Virol 2006 80 7 3617 3623 10.1128/JVI.80.7.3617-3623.2006 16537629 PMC1440414

[B43] Brockman MA Schneidewind A Lahaie M Schmidt A Miura T Desouza I Ryvkin F Derdeyn CA Allen S Hunter E Mulenga J Goepfert PA Walker BD Allen TM Escape and compensation from early HLA-B57-mediated cytotoxic T-lymphocyte pressure on human immunodeficiency virus type 1 Gag alter capsid interactions with cyclophilin A J Virol 2007 81 22 12608 12618 10.1128/JVI.01369-07 17728232 PMC2169025

[B44] Garcia JV Miller AD Serine phosphorylation-independent downregulation of cell-surface CD4 by nef Nature 1991 350 6318 508 511 10.1038/350508a0 2014052

[B45] Rossio JL Esser MT Suryanarayana K Schneider DK Bess JW Jr Vasquez GM Wiltrout TA Chertova E Grimes MK Sattentau Q Arthur LO Henderson LE Lifson JD Inactivation of human immunodeficiency virus type 1 infectivity with preservation of conformational and functional integrity of virion surface proteins J Virol 1998 72 10 7992 8001 9733838 10.1128/jvi.72.10.7992-8001.1998 PMC110135

[B46] Agosto LM Yu JJ Liszewski MK Baytop C Korokhov N Humeau LM O'Doherty U The CXCR4-tropic human immunodeficiency virus envelope promotes more-efficient gene delivery to resting CD4+ T cells than the vesicular stomatitis virus glycoprotein G envelope J Virol 2009 83 16 8153 8162 10.1128/JVI.00220-09 19493998 PMC2715791

[B47] Pierson TC Zhou Y Kieffer TL Ruff CT Buck C Siliciano RF Molecular characterization of preintegration latency in human immunodeficiency virus type 1 infection J Virol 2002 76 17 8518 8531 10.1128/JVI.76.17.8518-8513.2002 12163571 PMC136977

[B48] Spivak AM Salgado M Rabi SA O'Connell KA Blankson JN Circulating monocytes are not a major reservoir of HIV-1 in elite suppressors J Virol 2011 85 19 10399 10403 10.1128/JVI.05409-11 21795348 PMC3196433

[B49] Yang H Wu H Hancock G Clutton G Sande N Xu X Yan H Huang X Angus B Kuldanek K Fidler S Denny TN Birks J McMichael A Dorrell L Antiviral inhibitory capacity of CD8+ T cells predicts the rate of CD4+ T-cell decline in HIV-1 infection J Infect Dis 2012 206 4 552 561 10.1093/infdis/jis379 22711904 PMC4192045

[B50] Mendoza D Johnson SA Peterson BA Natarajan V Salgado M Dewar RL Burbelo PD Doria-Rose NA Graf EH Greenwald JH Hodge JN Thompson WL Cogliano NA Chairez CL Rehm CA Jones S Hallahan CW Kovacs JA Sereti I Sued O Peel SA O'Connell RJ O'Doherty U Chun T Connors M Migueles SA Comprehensive analysis of unique cases with extraordinary control over HIV replication Blood 2012 119 20 4645 4655 10.1182/blood-2011-10-381996 22490332 PMC3367872

[B51] Bailey JR Brennan TP O'Connell KA Siliciano RF Blankson JN Evidence of CD8+ T-cell-mediated selective pressure on human immunodeficiency virus type 1 nef in HLA-B*57+ elite suppressors J Virol 2009 83 1 88 97 10.1128/JVI.01958-08 18945771 PMC2612327

[B52] Bailey JR Williams TM Siliciano RF Blankson JN Maintenance of viral suppression in HIV-1-infected HLA-B*57+ elite suppressors despite CTL escape mutations J Exp Med 2006 203 5 1357 1369 10.1084/jem.20052319 16682496 PMC2121215

[B53] O'Connell KA Brennan TP Bailey JR Ray SC Siliciano RF Blankson JN Control of HIV-1 in elite suppressors despite ongoing replication and evolution in plasma virus J Virol 2010 84 14 7018 7028 10.1128/JVI.00548-10 20444904 PMC2898225

[B54] Salgado M Brennan TP O'Connell KA Bailey JR Ray SC Siliciano RF Blankson JN Evolution of the HIV-1 nef gene in HLA-B*57 positive elite suppressors Retrovirology 2010 7 94 10.1186/1742-4690-7-94 21059238 PMC2993647

[B55] Mens H Kearney M Wiegand A Shao W Schonning K Gerstoft J Obel N Maldarelli F Mellors JW Benfield T Coffin JM HIV-1 continues to replicate and evolve in patients with natural control of HIV infection J Virol 2010 84 24 12971 12981 10.1128/JVI.00387-10 20926564 PMC3004307

[B56] Zhang H Zhou Y Alcock C Kiefer T Monie D Siliciano J Li Q Pham P Cofrancesco J Persaud D Siliciano RF Novel single-cell-level phenotypic assay for residual drug susceptibility and reduced replication capacity of drug-resistant human immunodeficiency virus type 1 J Virol 2004 78 4 1718 1729 10.1128/JVI.78.4.1718-1729.2004 14747537 PMC369469

[B57] O'Connell KA Rabi SA Siliciano RF Blankson JN CD4+ T cells from elite suppressors are more susceptible to HIV-1 but produce fewer virions than cells from chronic progressors Proc Natl Acad Sci USA 2011 108 37 E689 98 10.1073/pnas.1108866108 21873218 PMC3174588

[B58] O'Doherty U Swiggard WJ Malim MH Human immunodeficiency virus type 1 spinoculation enhances infection through virus binding J Virol 2000 74 21 10074 10080 10.1128/JVI.74.21.10074-10080.2000 11024136 PMC102046

[B59] Rabi SA O'Connell KA Nikolaeva D Bailey JR Jilek BL Shen L Page KR Siliciano RF Blankson JN Unstimulated primary CD4+ T cells from HIV-1-positive elite suppressors are fully susceptible to HIV-1 entry and productive infection J Virol 2011 85 2 979 986 10.1128/JVI.01721-10 21068257 PMC3020020

