# Supplementary figures and images for: Primary CD8+ T cells from elite suppressors effectively eliminate non-productively HIV-1 infected resting and activated CD4+ T cells
Source: Retrovirology. 2013 Jul 1;10:68. doi: 10.1186/1742-4690-10-68 (PMC3702406; doi:10.1186/1742-4690-10-68)

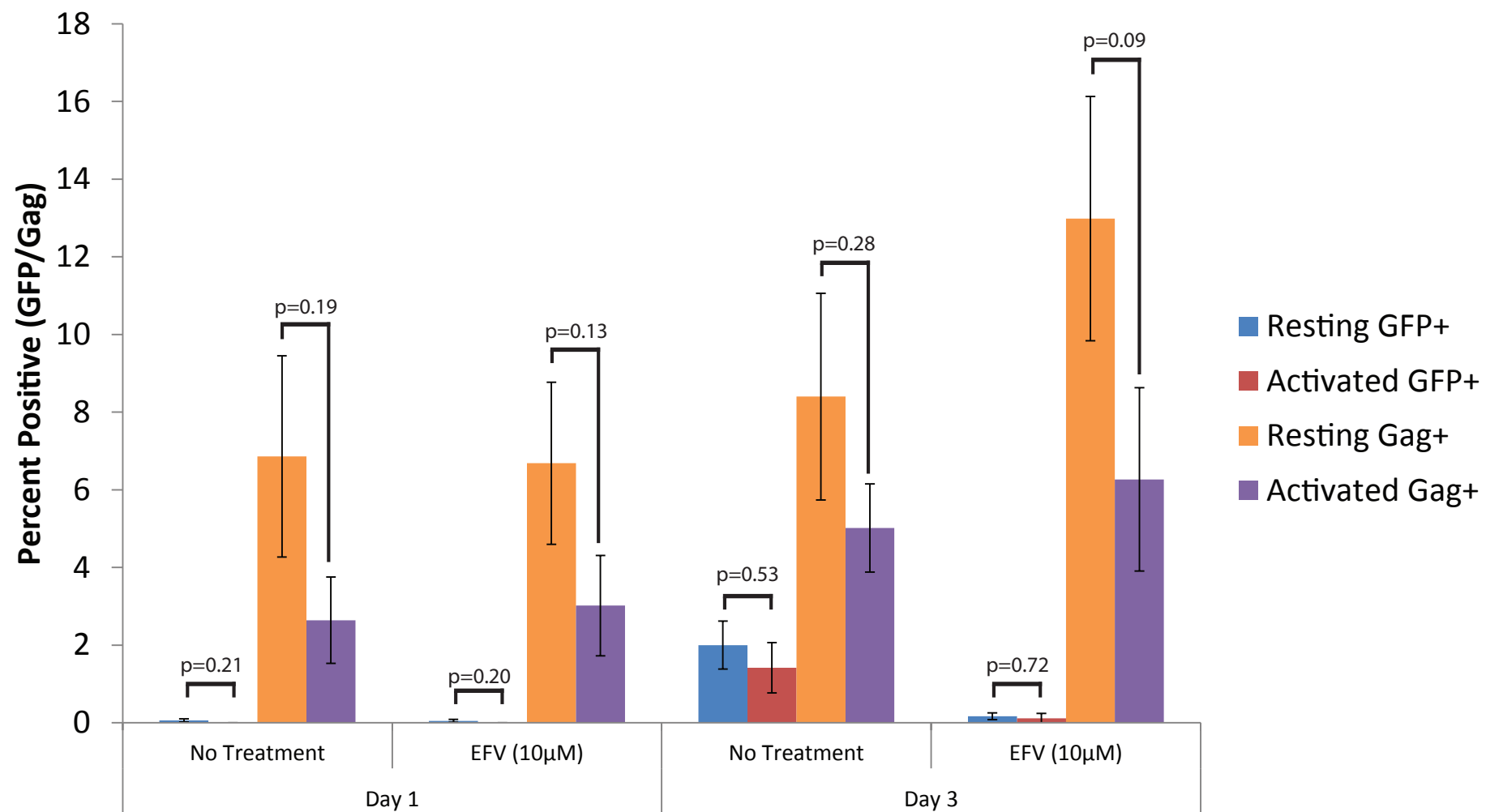

Supplement: Additional file 1 — Percent of Gag and GFP positive resting and activated cells present at 18 and 72 hours post infection in the presence or absence of EFV. [file 1742-4690-10-68-S1.pdf]
